# Types of Environmental Public Interest Litigation in China and Exploration of New Frontiers

**DOI:** 10.3390/ijerph20043273

**Published:** 2023-02-13

**Authors:** Wei You, Shan Liang, Lei Feng, Zexuan Cai

**Affiliations:** 1 Law School, Southwestern University of Finance and Economics, Chengdu 611130, China; 2 Law School, Sichuan University, Chengdu 610207, China; 3 The People’s Procuratorate of Chengdu Wenjiang, Chengdu 611130, China

**Keywords:** environmental public interest litigation, environmental civil public interest litigation, environmental administrative public interest litigation, types of environmental public interest litigation

## Abstract

Since “ecological civilization” was written into the constitution, China has continuously strengthened ecological and environmental protection and innovatively established an environmental public interest litigation system. However, China’s current environmental public interest litigation system is not sound, especially since the types and scope of environmental public interest litigation are unclear, which is the core problem we aim to solve. To explore the types of environmental public interest litigation in China and the possibility of expanding new fields, we first used the normative analysis method to review the legislation of environmental public interest litigation in China and then conducted an empirical analysis of 215 judgment documents of environmental public interest litigation in China, and we concluded that the legal types and scope of application of environmental public interest litigation in China are constantly expanding. To reduce environmental pollution and ecological damage as much as possible, we argue that China should further expand the application of environmental administrative public interest litigation to improve the environmental civil public interest litigation system and adhere to the criteria of “behavior standards first, result standards second” and “prevention first, recovery second”. At the same time, through the internal connection mechanism between procuratorial suggestions and environmental administrative public interest litigation, the external cooperation between environmental organizations, procuratorates, and environmental administrative departments should be strengthened, and a new mechanism for environmental public interest litigation should be established and improved to accumulate useful experience in the judicial protection of China’s ecological environment.

## 1. Introduction

Sustainable development has become the common pursuit of all countries in the world. All countries attach great importance to eco-environmental protection for a harmonious coexistence between human beings and nature. In recent years, China has attached great importance to ecological environmental protection. China incorporated ecological civilization into its constitution in the 2018 constitutional revision. With the support of the constitution, China’s ecological civilization construction has achieved certain results. However, given that China’s laws, policies, and environmental evaluation system are under continuous improvement, environmental problems in China have remained relatively prominent, and the overall deterioration of the ecological environment has not yet been fundamentally reversed. We illustrate in detail the frequent environmental pollution and ecological destruction in China in Section 3.2. In response, China has established an innovative environmental public interest litigation system to address these serious environmental and ecological damages. There is no doubt that China’s environmental public interest litigation system has played a positive role in improving China’s ecological environment and strengthening the construction of ecological civilization.

However, environmental public interest litigation in China remains in the exploratory stage, with an inadequate environmental public interest litigation system. Only phrases such as “pollution of the environment …… and other acts that harm the public interest of the society” and “pollution of the environment, destruction of the ecology, and harm to the public interest of the society ……” and “protection of the ecological environment and resources” are mentioned in the relevant laws, resulting in great controversy over the specific scope of environmental public interest litigation in China. In this regard, we focus on solving and answering this question. We try to explore the key types and new areas of environmental public interest litigation in China and build a reasonable environmental public interest litigation mechanism.

To resolve this question, Chinese academics have launched some discussions, mainly concerning the shape of environmental public interest litigation. For example, some scholars argued that China’s environmental civil public interest litigation was recognized in legislation but that environmental administrative public interest litigation was still in the authorized experimental stage and had not been involved in legislation [1]. For another example, some scholars even believe that China’s Civil Procedure Law has not established an environmental public interest litigation system and that the so-called “social public interest” is only a private interest [2]. Few studies, however, have explored the specific scope and field of environmental public interest litigation. Some scholars believe that the scope of environmental public interest litigation should be “illegal civil and administrative acts that seriously damage or threaten the public interest of the environment”, and the criteria for its identification should be clear [3]. However, they did not specify the criteria. Some scholars believe that the scope of environmental public interest litigation should include pre-litigation supervision of environmental administrative acts, compensation for ecological and environmental damage, cases transferred by environmental administrative departments or reported by the public, ecological restoration costs, and climate change [4]. However, the above scope does not cover the environmental public interest litigation brought by environmental organizations. Therefore, in this context, our study is of great significance. It answers the question of which fields and matters in China can be included in the scope of environmental public interest litigation in the future and provide guidance for constructing ecological civilization in China.

To answer this core question, we follow the following research paths and methods: First, we adopt a normative approach in this research to sort out and interpret Chinese legislative texts and judicial interpretations and try to reveal the normative expression of the types and scope of environmental public interest litigation in legislative texts, summarize the normative types, and explore the normative space. Second, we use an empirical analysis approach to organize and analyze adjudicative documents to objectively present what areas are involved in environmental public interest litigation in practice and to clarify the conflicts between these fields in practice and the provisions of the legislative text to reveal the contradictions between the norms and the reality. Third, based on the legislative texts and practice, we explore the key types and new areas of environmental public interest litigation in China and lay the foundation for constructing a reasonable environmental public interest litigation mechanism.

## 2. Legislative Evolution of Environmental Public Interest Litigation in China

Environmental public interest litigation has emerged in China under the appearance of public interest litigation. It has gradually become an important part of a diverse and distinctive legal system with Chinese characteristics through policy piloting, continuous regulation, and improvement in its content by laws and judicial interpretations, as well as innovation and institutional changes in practice.

### 2.1. Derived from Civil Public Interest Litigation: 2012–2014

Environmental public interest litigation is a branch of social public interest litigation in the field of environmental protection. It is clear from the normative changes in China’s legal system that environmental public interest litigation was derived from public interest litigation or social public interest litigation. The environmental public interest litigation system refers to the system in which specific state organs, relevant groups, and individuals file lawsuits against civil subjects or governments for infringing upon public environmental interests, and the court investigates the doer for legal liability [5]. The year 2012 saw the second amendment to the Civil Procedure Law since 2007, with a new article added as Article 55, which stipulates that the authorities and relevant organizations prescribed by law may file lawsuits in the people’s courts for acts that harm the public interest, such as pollution of the environment and infringement of the legitimate rights and interests of numerous consumers. The public interest litigation system was, therefore, formally established in Chinese law, which became a new starting point for the construction of the environmental public interest litigation system. The reason for establishing the public interest litigation system in the Civil Procedure Law and clearly listing two types of public interest litigation, environmental public interest litigation and consumer public interest litigation, is twofold: first, to solve the dilemma of the people’s courts accepting public interest litigation cases. Since 2007, environmental pollution has become a prominent problem in China. Despite this, most courts have not accepted such cases because they are not explicitly provided for by law, leading to public discontent. Although the Supreme People’s Court of China supported environmental public interest litigation by means of a normative document in 2010, the document has little guidance for judicial practice because of its low level of validity. Second, it responds to the needs of the Chinese people in terms of environmental protection and food safety. The majority of public interest litigation cases are environmental pollution and food safety accidents.

At this time, environmental public interest litigation is only a form of civil public interest litigation. It is included in the types of civil public interest litigation by way of enumeration, lacking its own clear type and scope. The amendment left the following questions open for discussion: What is the relationship between environmental public interest litigation and civil litigation? Is there any environmental administrative public interest litigation? How can we understand the term “acts detrimental to the public interest”, and does it mean that there are other types of environmental public interest litigation besides “pollution of the environment”?

### 2.2. The Foundation of the Environmental Protection Law and Related Judicial Interpretations: 2013–2014

The Environmental Protection Law, a special law for environmental protection in China, was enacted in 1979—the beginning of China’s economic reform. Currently, its amendment has been highly requested by various sectors of Chinese society as it no longer meets the requirements of China’s economic and social development. As a result, the Environmental Protection Law was amended for the first time in April 2014. However, environmental public interest litigation was not addressed in the Draft Amendment to the Environmental Protection Law. After the third meeting of the Standing Committee of the National People’s Congress, which held a second review of the draft amendments to the Environmental Protection Law, the environmental public interest litigation system received more attention. The Legal Committee considered that environmental public interest litigation, as a new system, should be actively and steadily promoted and eventually established in the Environmental Protection Law. Article 58 of the Environmental Protection Law stipulates that eligible social organizations may file lawsuits in the people’s courts against acts that pollute the environment or damage the ecology and harm the public interests of society and sets out the criteria for identifying social organizations. The amendment to the Environmental Protection Law expands the scope of application of environmental public interest litigation from “pollution of the environment” in the Civil Procedure Law to “pollution of the environment” and “damage to the ecology”.

However, the Environmental Protection Law and previous laws, such as the Civil Procedure Law, still adopt the principled provisions regarding the subject of litigation, the scope of accepting cases, and identifying evidence in environmental public interest litigation. The lack of specific and operable procedural rules resulted in the acceptance and trial of many cases without evidence. In December 2014, the Supreme People’s Court issued a judicial interpretation to accompany the Civil Procedure Law (amended in 2012) and the Environmental Protection Law (amended in 2014), which not only solved the problem of unclear specific rules in the acceptance and trial procedures but also reaffirmed that the scope of application of environmental public interest litigation covers both civil litigation arising from acts of pollution of the environment and litigation arising from acts of ecological damage. The amendment to the Environmental Protection Law not only applies to “acts of polluting the environment and damaging the ecology that have already harmed the public interest” but also “acts of polluting the environment and damaging the ecology that have a significant risk of harming the public interest” [6].

The amendment to the Environmental Protection Law means that environmental public interest litigation has advanced from a general law to a specialized law with a special judicial interpretation. The scope of application has been expanded from “polluting the environment” to “damaging the ecology” and from causing “existing damage” to “significant risk”. Unfortunately, the only type of environmental public interest litigation is civil environmental public interest litigation, and administrative environmental public interest litigation has not yet entered legal provisions.

### 2.3. Perfection in the Establishment of Procuratorial Public Interest Litigation: 2015–2020

In 2014, the Fourth Plenary Session of the 18th Central Committee of the Communist Party of China (CPC) studied several major issues in comprehensively promoting the rule of law and made a decision to optimize the configuration of judicial powers, requiring procuratorial organs to explore the establishment of a procuratorial public interest litigation system. In 2015, the 12th meeting of the Central Leading Group for Comprehensively Deepening Reform considered and adopted the Pilot Program for the Reform of Public Interest Litigation by Procuratorial Organs. The program requires procuratorial organs to file civil or administrative public interest litigation promptly to strengthen the protection of the state and social public interests in areas such as ecological environment and resource protection that infringe upon them. The pilot program also clarifies that the focus of administrative public interest litigation during the pilot period is the ecological environment and resource protection. The Civil Procedure Law and the Administrative Procedure Law, as amended in 2017, follow the provisions of the pilot program in Article 55(2) and Article 25(4), respectively, and specify the scope of application of environmental public interest litigation in the field of “ecological environment and resource protection”.

During this period, the Opinions on Issues Related to the Further Development of Public Interest Litigation Pilot Program issued by the Supreme People’s Procuratorate in December 2016 proposed, for the first time, to explore the form of criminal incidental civil public interest litigation. Furthermore, in 2017, civil public interest litigation incidental to environmental crimes began to emerge. In 2018, Article 20 of the Interpretation of the Supreme People’s Court and the Supreme People’s Procuratorate on Several Issues Concerning the Application of Law to Prosecution Public Interest Litigation Cases provided a normative basis for civil public interest litigation incidental to crimes.

The amendment enriched the prosecution subjects of environmental civil public interest litigation, filled the legislative gap of the lack of legal regulation of environmental administrative public interest litigation, and established the system of criminal incidental civil public interest litigation. At this point, environmental public interest litigation has been unimpeded in all three litigation channels of China’s socialist legal system [7]. Meanwhile, the Civil Procedure Law (amended in 2017), the Administrative Procedure Law (amended in 2017), and the Interpretation on Several Issues Concerning the Application of Law to Prosecution Public Interest Litigation Cases have included administrative violations and administrative inaction of administrative organs in the ecological environment and resource protection in the scope of application of environmental public interest litigation. The scope of application of environmental public interest litigation has been extended from civil subjects to administrative subjects, from positive acts of “pollution of the environment” and “damage of the ecology” to negative acts of administrative inaction, and from “pollution of the environment” and “damage of the ecology” to “ecological environment and resource protection”.

### 2.4. Expansion in the Establishment of the Compensation System for Ecological and Environmental Damages in the Civil Code: 2021–Present

The Third Plenary Session of the 18th CPC Central Committee studied several major issues of comprehensive deepening reform and proposed accelerating the construction of an ecological civilization system, requiring the implementation of a damage compensation system and the protection of ecology with the system. In 2017, the General Office of the CPC Central Committee and the General Office of the State Council issued the Reform Program of the Compensation System for Damage to the Ecological Environment. In response to the above policy requirements, the Civil Code establishes the green principle as the basic principle of civil law, and the tort liability part is modified to improve the ecological and environmental damage liability system, including the punitive compensation system for ecological and environmental damage and the repair and compensation system for ecological and environmental damage. However, the practice of filing both environmental public interest litigation and ecological damage compensation litigation against the same act has emerged successively, showing the confusion of the applicable law in judicial practice. The Supreme People’s Court of China then issued a judicial interpretation establishing the trial principle of “separate filing, combined trial, priority of ecological environmental public interest litigation”. It is specified that if the same act is filed in both ecological and environmental damage compensation lawsuits and civil public interest litigation, the people’s court that receives the ecological and environmental damage compensation lawsuit shall organize the trial based on separate cases and shall first suspend the trial of the civil public interest litigation case, and after the trial of the ecological and environmental damage compensation lawsuit is completed, make a decision on the litigation request that is not covered by the civil public interest litigation case according to law. After the trial of the ecological and environmental damage lawsuit is completed, it shall make a decision on the claims not covered by the civil public interest lawsuit in accordance with the law. Although the ecological environmental damage compensation system and the environmental public interest litigation system differ in legal application, some scholars believe that the two have theoretical implications and commonalities [8]. Some scholars even advocate that the essence of ecological and environmental compensation litigation is environmental civil public interest litigation, only a special kind of environmental civil public interest litigation [9]. If an ecological damage compensation lawsuit is regarded as a category of environmental public interest litigation, the scope of environmental public interest litigation will be extended to “ecological environmental damage”.

In summary, a review of China’s environmental public interest litigation legislation reveals that China’s environmental public interest litigation system has continued to improve with expanded prosecution subjects, legal types, and scope of application. This has effectively adapted to China’s policy development and practical needs. However, because of the continuous development of environmental public interest litigation in China, its legal types and scope of application are full of uncertainties. To accurately grasp the types and scope of environmental public interest litigation in China, we explore the expansion of relevant organizations and procuratorates into new areas of environmental public interest litigation through the combination and analysis of adjudicative documents in the following chapters.

## 3. The Practice of Environmental Public Interest Litigation in China

Environmental public interest litigation adjudication documents are valuable samples that reflect the current status and problems of China’s environmental public interest litigation system. We searched the full text of 410 environmental public interest litigation cases from 2012 to 2021 on the China Judicial Documents Website and obtained a total of 215 valid environmental public interest litigation cases by eliminating duplicate and irrelevant cases. By analyzing these documents, we can generally grasp the characteristics and problems of the current environmental public interest litigation system in China.

### 3.1. Litigation form of Environmental Public Interest Litigation in China

Currently, Chinese scholars generally believe that China has established an environmental public interest litigation system. However, with regard to the litigation form of environmental public interest litigation, many scholars believe that environmental public interest litigation in China mainly refers to environmental civil public interest litigation rather than environmental administrative public interest litigation. In other words, many scholars believe that China has not established environmental administrative public interest litigation [10]. Through the analysis of judgment documents, we find that there are mainly two types of environmental public interest litigation in China’s judicial practice, showing two distinct characteristics.

#### 3.1.1. Environmental Public Interest Litigation in China Is Dominated by Environmental Civil Public Interest Litigation

By sorting out the path of environmental public interest litigation in Chinese legislation, we can see that the types of environmental public interest litigation in China are closely related to the three types of litigation in China, which can also form three types of environmental civil public interest litigation, environmental administrative public interest litigation, and environmental criminal incidental civil public interest litigation. So-called environmental civil public interest litigation refers to litigation in which citizens or organizations request the court to provide civil relief for the acts of other citizens or organizations infringing on public environmental interests. So-called environmental administrative public interest litigation refers to a judicial review lawsuit filed by a citizen or legal person to the court on the grounds that the specific environmental administrative acts of governments are damaging public environmental interest [11]. The main difference between the two lies in the objects. Environmental civil public interest litigation mainly targets the behaviors of private individuals that pollute the environment or destroy the ecology, while environmental administrative public interest litigation mainly targets the behaviors of governments.

In fact, among the 215 environmental public interest litigation decisions, there are 157 environmental civil public interest litigations and environmental administrative public interest litigations and 45 environmental criminal public interest litigations. It can be seen that environmental public interest litigation, which was born out of civil public interest litigation, is still dominated by environmental civil public interest litigation. At the same time, the judicial practice has also dispelled scholars’ doubts about the existence of administrative environmental public interest litigation in China. Although the current environmental public interest litigation in China is mainly civil public interest litigation, there has been an increase in administrative public interest litigation brought by administrative organs for failure to perform their statutory duties or administrative violations in recent years.

#### 3.1.2. The Main Subjects of Environmental Public Interest Litigation in China Are Environmental Organizations and Procuratorates

Before environmental public interest litigation was stipulated in the law, scholars carried out many discussions on the types of plaintiffs in environmental public interest litigation. For example, some scholars believe that citizens, social groups, and procuratorial organs can be plaintiffs in environmental public interest litigation, but environmental administrative organs are not allowed to file lawsuits [7]. Some scholars also believe that the subjects of environmental public interest litigation should include procuratorates, state environmental administrative organs, social groups, citizens, and future generations [12]. Since China’s Civil Procedure Law, Administrative Procedure Law, and Environmental Protection Law limited the subjects of environmental litigation to the people’s procuratorates and related organizations, the focus of Chinese academic research has shifted to how to give full play to the role of these two subjects. According to the analysis of 215 environmental public interest lawsuits, there are 109 environmental public interest lawsuits filed by procuratorates in China, including 51 civil public interest lawsuits, 13 administrative public interest lawsuits, and 45 criminal public interest lawsuits. There are 106 environmental public interest lawsuits filed by related organizations. This shows that environmental protection administrative departments and organizations specializing in environmental protection public interest activities, jointly with the procuratorate, effectively shoulder the responsibility of safeguarding public interest in practice. In addition, the main subjects of environmental public interest litigation prosecution are relevant organizations and procuratorates, which is consistent with China’s legislation on environmental public interest litigation.

It is worth noting that the two types of lawsuits, environmental civil public interest litigation and environmental administrative public interest litigation, are defined only by the nature of the relationship between the subjects of the lawsuit and the nature of responsibility without any matter-based provisions. In other words, we still need to examine the matter of environmental public interest litigation.

### 3.2. The Scope of Environmental Public Interest Litigation in China

In terms of the scope of environmental public interest litigation, China’s current legislation stipulates that environmental public interest litigation applies to “pollution of the environment”, “damage to the ecology”, “damage to the ecological environment and resource protection”, and other areas. To be specific, civil environmental public interest litigation applies to “pollution of environment” and “damage to ecology”, while administrative environmental public interest litigation and civil environmental criminal public interest litigation apply to “damage to ecological environment and resource protection”. However, the existing legislation in China does not specify the criteria for determining whether an act is “polluting the environment” or “damaging the ecology”, but rather mainly leaves it to practice. This has indirectly led to environmental public interest litigation involving various fields. According to an analysis of 215 environmental public interest lawsuits, China’s environmental public interest lawsuits mainly focus on the following 5 fields.

#### 3.2.1. The Field of Water Resources

In practice, citizens, legal persons, or other organizations have caused water pollution by secret pipes, cross-dumping, illegal disposal of pollutants, and erection of illegal buildings, which have led to environmental public interest litigation. Such cases account for a relatively large proportion in Chinese practice. In the field of water pollution, the following behaviors are mainly manifested: The first is the illegal construction of farms. For example, the People’s Procuratorate of Shiyan City, Hubei Province, and the water pollution liability environmental public interest litigation case of a pig farming cooperative in Yunxi County, where the defendant illegally built a farm and discharged excrement from the farmed pigs directly into the river, resulting in foul-smelling river water, deteriorating water quality, and loss of function, causing serious impacts on local residents and the local government [13]. The second concerns the dumping of pollutants. For example, in the case of Jiangsu Provincial People’s Government against Anhui Hyde Chemical Technology Co., Ltd. (Maanshan, China) for ecological and environmental damage, the defendant handed over the waste lye produced in the production process to individuals who were not qualified to dispose of it. This was considered by the court as inaction causing environmental pollution damage [14]. Another form of water pollution is caused by illegal or over-standard discharge. For example, in the case of Chongqing Liangjiang Volunteer Service Development Center and Pingxiang Pinggang Anyuan Iron and Steel Co., Ltd. (Pingxiang, China) and Pingxiang Baohai Zinc Nutrition Technology Co., Ltd.’s (Pingxiang, China) environmental pollution liability dispute, the defendant failed to build and improve the sewage diversion system and illegally discharged pollutants, causing water pollution [15]. Finally, water pollution can be based on accidental events. When the defendant conducted blasting operations, the silt and pollutants accumulated in the reservoir for years washed downstream, polluting the water source [16]. Another example is the Quzhou Communication Logistics Co., Ltd. (Quzhou, China) and Ping An of China Property Insurance Co., Ltd. (Shenzhen, China) Quzhou Central Branch Company Environmental Pollution Liability Dispute, in which the defendant caused serious pollution of water resources due to the overturning of a vehicle and the leakage of hydrofluoric acid from the vehicle flowing into the drainage ditch [17].

#### 3.2.2. The Field of the Atmosphere

Under the concept of green, low-carbon cycle development, China focuses on environmental public interest litigation against coal burning, industrial pollution, motor vehicle and vessel pollution, dust pollution, agricultural pollution, and other acts that damage the atmosphere. The first manifestation is the illegal or excessive emission of pollutants into the atmosphere. For example, in the public interest litigation case between Jiangsu Daji Power Generation Co., Ltd. (Yancheng, China) and Beijing Chaoyang District Friends of Nature Environmental Research Institute on air pollution liability disputes, the defendant over-emitted sulfur dioxide, nitrogen oxides, and particulate matter into the atmosphere during waste incineration, causing pollution and damage to the ecosystem [18]. The second form is motorboat exhaust pollution. For example, in the case of China Biodiversity Conservation and Green Development Foundation and Shenzhen Speedy Environmental Protection Co., Ltd. (Shenzhen, China) and Zhejiang Taobao Network Co., Ltd.’s (Hangzhou, China) air pollution liability dispute, the defendant sold purifiers by falsification to help vehicles with excessive tailpipe emissions pass the annual inspection, causing extremely serious impacts on China’s air pollution prevention and control [19]. The third form concerns the burning of pollutants. For example, in the case of Lai’s pollution of the environment, the defendant illegally disposed of hazardous waste and excessively burned waste circuit board components, which seriously polluted the environment [20].

#### 3.2.3. The Field of Biodiversity

As the country with the richest biodiversity in the world, disputes over wildlife protection, fisheries and forestry resources protection, animal and plant quarantine, and new plant varieties have occurred frequently, among which wildlife hunting cases in particular account for a relatively large proportion. Such cases endanger rare animals and plants and their living environment and have attracted widespread attention in judicial practice. In the field of biodiversity, the main types of cases are as follows: The first type concerns illegal hunting, smuggling, and trafficking of wild animals and animal products. For example, in the case of the ecological environment infringement dispute between Songyuan City People’s Procuratorate of Jilin Province and Yu, the defendant Yu used illegal hunting tools to catch wild birds with important ecological, scientific, and social values in his own yard, which damaged national wildlife resources and destroyed the stability of the ecosystem. As a result, Yu was sentenced by the court to pay compensation for ecological environment restoration costs and deliver a public apology [21]. The second type concerns illegal fishing of aquatic products. The civil public interest lawsuit against Wang and 59 others for ecological damage brought by the People’s Procuratorate of Taizhou City, Jiangsu Province, is the first case in China in which the acquirer and the illegal fisherman were ordered to bear several joint liabilities for ecological resource loss caused by joint infringement under the condition of a fixed buying and selling relationship and a complete interest chain. The case is an innovative way of assuming liability by offsetting the amount of compensation by labor substitution. It allows the defendant to participate in activities of a public interest nature, such as protecting the ecological environment of the Yangtze River or cooperating in management, reinforcement, and garbage cleanup along the Yangtze River to offset the amount of compensation [22]. The final type concerns the destruction of biological habitats. For example, in the case of Zunyi City People’s Procuratorate v. Xiao Moukai and Xiao Moubo, a civil public interest lawsuit for ecological and environmental damage, the court held that the two defendants had illegally occupied the mountain forest land and dark river caves without obtaining legal land use and other approval procedures to illegally build a lodge, which destroyed the unique environmental function and ecological value of the cave. This is because caves, as a typical representative of karst geomorphology in Guizhou Province, are small miniature ecosystems composed of various plants and animals; karst can be found in caves, dark rivers, and other environmental elements, as well as natural habitats, and they are growing places for snakes, insects, bats, fish, and shrimps, which have unique ecological and environmental values [23].

#### 3.2.4. The Field of Resource Development

In the field of resource development, there is the phenomenon of over-exploitation of natural resources such as grasslands, forests, mineral deposits, and marine lakes, which affects the protection and development of natural resources and contradicts the concept of sustainable development. The main manifestations are as follows: The first manifestation is illegal mining and sand mining cases. For example, in the tort liability dispute between Fujian Green Home Environment Friendly Center and Dingyu Building Materials Co., Ltd. (Zhangzhou, China), Dingyu exceeded the approved scope of the mining license in the process of mining ore resources, which caused damage to the ecological environment [24]. The second type is illegal land reclamation cases. For example, in the case of the public interest litigation prosecutor Jilin City People’s Procuratorate v. Chen environmental pollution liability disputes, Chen carried out illegal reclamation in his contracted grassland and planted crops, which destroyed grassland vegetation. As grasslands play an important role in regulating regional climate, increasing rainfall, reducing sandy weather, and maintaining regional ecological balance, this act constitutes damage to the ecological environment of grasslands [25]. The third type is illegal deforestation and grass digging. For example, in the third branch of Chongqing People’s Procuratorate v. Zhang environmental damage compensation public interest litigation case, Zhang illegally occupied public welfare forest land and destroyed the original woody trees on the ground, destroying forest land, increasing the risk of geological disasters, and causing serious damage to the ecological environment [26]. The final type is illegal lake reclamation. For example, in the case of Beihai Nai Zhi Marine Technology Co., Ltd. v. Beihai City Bureau of Ocean and Fisheries Administrative Penalty, without obtaining the legal right to use the relevant sea area, Nai Zhi carried out sea enclosure and reclamation in the area, which constituted illegal sea enclosure and reclamation. Its act changed the natural properties of the sea area and formed a land area, which harmed the offshore ecology [27].

#### 3.2.5. The Field of Cultural Heritage

In judicial practice, there are not only cases of environmental rights and interests of the masses arising from the traditional dwellings, ancient villages, and ancient buildings of individual residents, but also cases of environmental infringement arising from cultural heritage sites, as well as cases of environmental restoration of water and soil erosion, land sanding and rock desertification in nature reserves, etc. The main manifestations are as follows: The first is the destruction of cultural heritage sites. For example, in the case of Xuedian Town People’s Government of Xinzheng City and China Biodiversity Conservation and Green Development Fund and other environmental civil public interest litigation, the defendant transplanted and caused the death of ancient jujube trees without going through logging and transplanting procedures according to the law, which was considered to have caused ecological damage and harmed historical and humanistic resources [28]. The second type concerns the damage to ecological reserves. For example, in the case of Wudang District Water Affairs Bureau in Guiyang City, the defendant constructed buildings on the banks of the Pudu River for business activities without authorization, which led to the destruction of water resources and the native river channel in the Pudu River basin and affected the natural ecology of the river [29]. The final type is the destruction of natural scenery. For example, in the case of Zhang and other ecological damage civil public interest litigation, the defendant used an electric drill to drill holes in the body of a rock pillar in a world-class natural relic, the Monty Python Peak, and then drove in rock nails so that the nails were firmly embedded in the body of the pillar for climbing, causing damage to the Monty Python Peak. The court held that natural relics and scenic spots are a kind of environmental element with originality and scarcity characteristics, which are difficult to restore once damaged. The destruction of natural relics and scenic landscapes not only damages the common property rights and interests of the country’s people but also impairs the rights and interests of the public in the recreation and viewing of unique scenery enjoyed by natural relics and scenic landscapes [30].

In addition to the above fields, matters of environmental public interest litigation in China involve soil pollution, noise pollution, and solid waste caused by above-ground water, and groundwater pollution cases also occur occasionally. In general, the scope of environmental public interest litigation in judicial practice is divided into two main types: pollution of the environment and ecological damage. The concept behind the pollution of the environment is the pursuit of “blue sky, blue water and clean land”, which is mainly manifested in water, atmosphere, soil, and noise pollution. Ecological damage is mainly manifested in illegal or excessive fishing, wildlife trafficking, illegal or excessive cutting of plants, destruction of wildlife habitats, and illegal or excessive reclamation of land, grassland, mineral deposits, forests, sea areas, and other natural resources. The concept behind which is to systematically protect precious wildlife and their living environment, maintain biodiversity, and achieve rational exploitation of natural resources.

### 3.3. “Performative Contradiction” in Environmental Public Interest Litigation in China

With the inclusion of “ecological civilization” in China’s constitution and the joint efforts of the theoretical and practical circles, China’s environmental protection work has made obvious progress. However, given that the current environmental public interest litigation system is under continuous improvement, the practice of environmental judicial protection also suffers from a lack of understanding of the concept and the insufficient role of specialized trial organizations. Environmental public interest litigation in China also faces a great contradiction between theory and practice.

#### 3.3.1. Environmental Administrative Public Interest Litigation Faces the Dilemma of Weak Normative Effectiveness

Article 25 of China’s Administrative Litigation Law stipulates that the people’s procuratorate shall, in the course of performing its duties, put forward procuratorial suggestions to the administrative organs when discovering administrative violations or omissions in the field of ecological environment and resource protection. Only if the administrative organs do not perform their duties in accordance with the law shall it institute environmental administrative public interest litigation. Therefore, Chinese academics generally believe that after the pilot program and the law, China has established environmental administrative public interest litigation. This is significant in urging environmental administrative organs to actively perform their legal duties. However, in practice, when there are acts of environmental pollution and ecological damage, the people’s procuratorate usually sends procuratorial suggestions to the administrative organs first. This is an important way for the procuratorate to perform its procuratorial functions and is non-compulsory [31]. In addition, it is easy for the procuratorate to judge the abuse of authority and overstepping of authority by the administrative organs, but there is no uniform standard for the administrative organs to perform their statutory duties or not to perform their statutory duties completely [32]. After the issuance of procuratorial recommendations, it is difficult for the administrative organs to determine between “should act but not act” and “should act but not fully act”, which means most of the environmental pollution and ecological damage cannot be carried out in terms of environmental administrative public interest litigation and greatly limits the scope when applying environmental public interest litigation. This is the reason why we found that the application rate of environmental administrative public interest litigation is not high when combing judicial documents.

#### 3.3.2. The Criteria for Determining the Scope of Environmental Public Interest Litigation Are Vague

China’s existing legislation only mentions “pollution of the environment” and “damage to the ecology” without specifying them. This has led to a variety of standards adopted by the courts in judicial practice, and the involved parties are unable to state the concept and boundaries between “pollution of the environment” and “damage to the ecology”. Due to the lack of criteria for applying environmental public interest litigation, it is easy to include cases that do not belong to environmental public interest litigation in the judicial field, adding additional judicial costs. Moreover, it is easy to exclude cases that should belong to environmental public interest litigation. For example, in the aforementioned case of Zhang and other ecological damage civil public interest litigation, the court distinguished environmental tort cases into two categories, environmental pollution and ecological damage, but ultimately did not specify which of the above two categories the case belonged to, and only stated in general terms that the case was an infringement of public social interest. Another example is that in the case of China Green Development Association v. the Housing and Urban-Rural Construction Bureau of Qingjiang District, Huai’an City; the plaintiff Green Development Association interpreted “ancient dwellings” as “environment” and the defendant’s “destruction of ancient dwellings” as “ecological damage”, showing the unclear scope of environmental public interest litigation in practice [33].

In fact, “pollution of the environment” and “damage to the ecology” are two distinct concepts. “Pollution of the environment” was first defined by the OECD Environment Committee as the direct or indirect entry of substances or energy into the environment by people, resulting in harmful effects on nature that threaten human health, endanger living resources and ecosystems, and impair or impede comfort and other legitimate uses of the environment [34], such as pollution by exhaust gases, waste water, sludge, and dust. “Damage to the ecology” mainly refers to the adverse consequences of exploiting natural resources. This includes the destruction of forests, water, and soil; desertification; overfishing; and biological extinction caused by human activities, which are distinctly different from environmental pollution [35]. However, some scholars propose that there are three main types of environmental public interest litigation: one is the pollution of water, gas, and other environmental elements; the second is the destruction of natural resources such as forests and minerals; and the third is damage to ecosystems such as wetlands and species [36]. Obviously, this enumeration scheme cannot cover all types of environmental public interest litigation in practice. Due to the lack of unified criteria for determining “pollution of the environment” and “damage to the ecology”, the public prosecutor’s office and related organizations generally do not choose to file public interest litigation against ambiguous acts of pollution and damage to the ecology, resulting in a great limitation to the scope of applying environmental public interest litigation.

#### 3.3.3. There Are Conflicts and Difficulties in the System of Environmental Public Interest Litigation

Environmental public interest litigation is a huge and complicated system. Therefore, the internal interface of the environmental public interest litigation system and the external interface between the environmental public interest litigation system and other systems are particularly important for the overall function of environmental public interest litigation. However, at present, China’s environmental public interest litigation has its own internal system conflict and insufficient connection with the external system.

First, the scope of environmental civil public interest litigation by the people’s procuratorate is narrower than that of the people’s court [37]. Specifically, the people’s procuratorate can only initiate environmental civil public interest litigation for acts that have caused damage to the ecological environment and resource protection, while the people’s court can accept environmental public interest litigation for acts that have caused damage to the public interest. The scope of environmental public interest litigation is “the act of polluting the environment or damaging the ecology that has already damaged the public interest or has a significant risk of damaging the public interest”, which includes the type of preventive environmental public interest litigation and has a broader scope. This internal conflict also greatly restricts the application of environmental public interest litigation. Through the collation and analysis of judicial documents, we also found that, in practice, environmental public interest litigation mainly tends to litigate the damage that has already occurred but ignores the risk of preventive environmental public interest litigation.

Second, as far as the external interface system is concerned, there are two problems. The first is that the interface between environmental administrative public interest litigation and environmental resource crimes is prominent. The Criminal Law of the People’s Republic of China is mostly a blanket crime of violating relevant environmental laws and regulations, which is one of the elements of environmental crimes, reflecting the strong subordination of environmental laws and regulations. However, environmental public interest litigation has the disadvantage of lacking matter provisions in the scope of application, which directly affects the identification of environmental resource-based crime [38]. The second problem is the lack of interface between the environmental public interest litigation system and the special litigation system. For example, in marine environmental claims litigation, the litigation procedure between marine environmental claims litigation and environmental administrative public interest is not well-connected. The difference between these two types of cases in terms of filing standards and acceptance difficulty is large. Identifying the negative administrative action of the marine environmental regulatory department and the causal relationship between the negative administrative action and the marine environmental pollution in the environmental administrative public interest litigation has become a problem in the filing stage. For example, marine environmental lawsuits can only be filed if they “have caused significant damage”, which is incompatible with the scope of environmental public interest litigation, which includes preventive environmental public interest litigation [39].

In summary, through the empirical analysis of judicial decisions on environmental public interest litigation, it is found that China’s environmental public interest litigation currently prefers to apply to environmental civil public interest litigation, while environmental administrative public interest litigation still has many difficulties due to the legislative design. At the same time, Chinese legislation does not clarify the specific meaning of “pollution of the environment” and “damage to the ecology”, which leads to the limitation of environmental public interest litigation in judicial practice to more obvious and easily judged areas. This undoubtedly limits the scope of the application of environmental public interest litigation.

## 4. Expansion of the Scope of Environmental Public Interest Litigation in China

There is still a gap between the requirements of China’s new era of ecological civilization and the people’s judicial needs for a beautiful ecological environment. To strengthen the judicial protection of ecological civilization, it is necessary to reform and innovate the established environmental judicial mechanism. Through the legislative review and judicial examination of environmental public interest litigation in China, it can be observed that the current field of environmental public interest litigation in China has yet to establish a clear identification standard. The only way to better serve China’s high-quality economic and social development is to expand the types and scope of environmental public interest litigation according to the law.

### 4.1. Theoretical Basis for the Expansion of New Areas of Environmental Public Interest Litigation in China

#### 4.1.1. China’s Inspiration from Other Countries’ Theories and Judicial Systems

First is the public trust theory. The theoretical basis of environmental public interest litigation can be traced back to the ancient Roman era. In Roman law, the common property of mankind, such as air, sunlight, and sea, was considered to be held in trust by the king or the government for public interest and public use, thereby serving as the origin of the public trust theory. The public trust theory was formally applied to environmental law as a result of further development by Joseph L. Sax of the University of Michigan in his scholarly dissertation summarizing the traditional public trust theory and then extending it to the field of natural resources. [40] Since then, the environmental public trust theory has been formalized as the foundation theory of the environmental law discipline [41]. The U.S. citizen suit system was developed under the environmental public trust doctrine, whereby a citizen or environmental NGO that has notified a government environmental enforcement agency of an environmental violation can sue the polluter as a plaintiff on behalf of the public interest if the environmental enforcement agency fails to take any action on the environmental violation within a certain period of time [42]. The “public interest trust theory” expands the object of environmental public interest litigation from ordinary torts, i.e., infringement of state and collective property rights, to the inaction of government agencies, which provides the theoretical basis for establishing administrative environmental public interest litigation in China.

Second is the private attorney general doctrine. The private attorney general doctrine originated in England is also considered to be an important rationale for the establishment of the citizen suit system in the United States [43]. The private attorney general system means that the Federal Assembly can grant the attorney general the right to prosecute in accordance with the Constitution to prevent public officials from engaging in acts that violate the authority granted by law. The attorney general can be either an official or a non-official as long as it is for the public good. This shows that the purpose of this system is to protect the public interest only, and the person who accepts this authorization is the private attorney general [44]. The private attorney general doctrine authorizes the attorney general to stop illegal acts of officials, which provides a theoretical basis for the plaintiff types of environmental public interest litigation in China to be divided into procuratorial organs and environmental protection organizations.

Third is the theory of environmental rights. Environmental rights are regarded as the third generation of human rights in China. The concept of environmental rights mainly originated in developed countries such as Europe and America. It provides a theoretical basis for citizens to enjoy the right to live in a good environment. As recognized by the academic community, since citizens have rights to the environment, when such rights are materially infringed, citizens should be entitled to defend their rights through litigation. The theory of environmental rights is widely favored in developed countries such as the United States, and China is no exception. Since the concept of ecological civilization was first put forward in the report of the 17th National Congress of the Communist Party of China, China has been deepening the institutional reform of ecological civilization, and it has been written into the constitution. Ecological civilization has been elevated from a political concept to a legal concept, and it has become an indisputable fact that citizens enjoy environmental rights in the constitution [45]. Obviously, the environmental rights theory has a great influence on China, and China’s environmental public interest litigation system is mainly based on the environmental rights theory.

#### 4.1.2. The Theory of Environmental Rights Has Been Recognized and Expressed in Chinese Law

The theory of environmental rights has been recognized and implemented by the international treaties China has acceded to. In 2021, China attended the World Congress on Environmental Justice as the 15th Party to the Convention on Biological Diversity. The meeting adopted the Kunming Declaration of the World Congress on Environmental Justice, which clarified that environmental justice should adhere to the principles of fairness, protection, and sustainable use of natural resources. The principles and concepts of international environmental treaties have been incorporated into China’s judicial system through China’s environmental judicial adjudication. The trial of such typical cases as the “green peacock protection case” shows that China’s environmental courts protect international environmental rights and interests and promote community building with a shared future through justice. On 6 November, China adopted the Wuhan Declaration on wetland protection, marking the Chinese government’s firm determination to strengthen wetland protection. It can be seen that both the Chinese judicial organs and the Chinese government have provided a sufficient institutional guarantee for the incorporation of international environmental treaties into China’s judicial system.

Under the influence of international treaties and other state theories and systems, Article 26 of the Constitution of China establishes the state’s obligation to protect and improve the ecological environment and to prevent pollution, which satisfies the expectation that “environmental rights are constitutionalized” by imposing different levels of obligations on the state [46]. According to other scholars, Article 33(3) of the constitution, “The State shall respect and guarantee human rights”, can also be deduced from the constitutional right to the environment as a subjective right [47]. Another example is that Article 9 of the Civil Code establishes the green principle of “conservation of resources and protection of the ecological environment”, and through Articles 1234 and 1235 of the Civil Code, the environmental interests of an unspecified majority are recognized and protected, transforming the abstract environmental rights into concrete and operable norms and realizing the civil law expression of environmental rights [48].

#### 4.1.3. The Theory of Environmental Rights Can Be Used to Explain the Cases in Chinese Judicial Practice

The environmental problems caused by “man-made activities” can have two kinds of related damage results: first, the result of overall “environmental damage”; and later, the result of the overall “environmental damage” and then specific “damage to property or physical health of others”. The former is regulated and remedied by the Environmental Protection Act, while the latter is remedied by the Tort Liability Act [49]. The former is a remedy for the collective environmental rights of human beings, while the latter is a remedy for the personal or property interests of a specific individual or several specific individuals. The former involves the public interest of society, while the latter is merely a collection of the private interests of some individuals. In the previous review of cases in practice, the application of environmental public interest litigation cases applies to water, atmosphere, soil, mineral deposits, forests, and other environmental and ecological elements common to all human beings rather than to the personal or property interests of individuals. This shows the boundary between environmental public interest litigation cases and traditional tort litigation cases caused by the environment.

On the morning of 11 January 2023, the Supreme People’s Court of China held a press conference on the special Guiding Cases of environmental public interest litigation and released the 37th batch of guiding cases of the Supreme People’s Court. Up to now, the Supreme People’s Court of China has issued 25 guiding cases and 116 typical environmental public interest litigation cases. These guiding cases and typical cases effectively play the role of case demonstration and rule supplement and fully reveal the possibility of the application of “the theory of environmental rights” in China’s judicial practice. For example, in Guiding Case No. 128 issued by the Supreme People’s Court, it is clear that individuals enjoy environmental rights and interests. The court held that the strong light generated by the defendant’s use of an LED display to play advertisements and promotional materials was obviously beyond the tolerable degree of ordinary people, which constituted damage to the environmental rights and interests of the plaintiff and others. Therefore, it is an objective fact that the plaintiff’s life is disturbed by light pollution and environmental rights and interests are harmed, even though the plaintiff has not developed obvious physical diseases [50]. As another example, in Guidance Case No. 208 issued by the Supreme People’s Court, the court directly explained what environmental rights and interests are. The court held that the damage to Monty Python Peak caused by the three people who climbed the mountain by inserting climbing pegs was against the environmental rights and interests of the non-specific social public, and the interests enjoyed by the non-specific majority were the connotation of public social interests. The environmental rights and interests enjoyed by people not only include the basic environmental elements necessary for survival and development, such as fresh air and clean water, but also include the ecological environmental resources generated based on the environment that can meet people’s higher needs, such as beautiful scenery, endangered animals of great scientific research value, or scarce plants or natural resources of significance ecological protection. The damage to these resources directly damages the natural nature and diversity of the ecological environment that people can feel and even produces ecological risks that people cannot feel in a short time.

It can be seen that the theory of environmental rights can provide theoretical support for us to clarify the scope and specific matters of environmental public interest litigation. In other words, environmental public interest litigation is litigation caused by infringement of environmental rights, and the objects of environmental rights—such as air, water, sunlight, and other environmental elements necessary for human beings—are the objects of environmental public interest litigation [51].

### 4.2. Key Types and Standards of New Areas of Environmental Public Interest Litigation in China

In the face of the application difficulties of environmental public interest litigation in new fields, it is easy to take the environmental rights theory as guidance, the protection of the object of environmental rights, and other specific environmental and ecological factors as the primary goal of environmental public interest litigation. The protection of specific environmental and ecological elements is often achieved through prohibiting illegal acts and remedying damage. Therefore, when refining the criteria for the expansion of environmental public interest litigation into new areas, the primary task is to determine the type of environmental public interest litigation to focus on, followed by the need to choose between prohibiting illegal acts and repairing the consequences of damages and, finally, to judge between the goal of prevention beforehand and the goal of relief afterward.

#### 4.2.1. The Study Should Focus on the Important Type of Environmental Administrative Public Interest Litigation

The focus of environmental administrative public interest litigation is to urge local governments and environmental protection departments to perform their duties. Meanwhile, environmental civil public interest litigation is taken to stop and punish private violations of the environment, and its implementation depends on the enthusiasm of social organizations. Therefore, environmental administrative public interest litigation is the development direction and focus of environmental public interest litigation, and environmental administrative public interest litigation should occupy a dominant position in environmental public interest litigation. The main reasons are as follows: First, the government is the first body responsible for maintaining environmental public welfare. Considering the government has a special constitutional and legal mandate to protect the environment and manage the environment, it should be supervised by the people. Second, local governments are more comprehensive and professional. This is because the local government can combine the enforcement capacities of various departments to improve environmental management. Moreover, professional ability is reflected in the government’s environmental protection departments in daily law enforcement, such as testing reports, assessment reports, and other first-hand professional materials, which can be directly presumed as valid evidence in subsequent litigation, greatly reducing the cost of litigation [52]. Third, there is a lack of motivation and incentives for social organizations to file civil environmental lawsuits. In judicial practice, even though social organizations are legally qualified to file environmental public interest litigation, they still show a low level of motivation. Some scholars believe that the lack of motivation for social organizations to file environmental public interest lawsuits is due to financial and work pressure and the lack of an incentive system for plaintiffs to win [53]. Therefore, the superiority and rationality of the environmental administrative public interest system is the focal point for the continuous innovation of China’s future environmental protection judicial system.

#### 4.2.2. The Scope of Environmental Public Interest Litigation Should Be Based on the Principle of “Behavior Standard as the Main Criterion and Result Standard as a Supplement”

The “illegal acts” of the administrative organs should be the main focus of environmental public interest litigation, and the “damage” caused by the infringers should be the secondary focus of environmental public interest litigation. This system contains two basic elements: first, the system adopts a binary standard, which means that both illegal acts and damages belong to the scope of application of environmental public interest litigation; second, as far as the system is concerned, there is a skewed allocation of judicial resources, i.e., more emphasis is placed on how “illegal acts” are included in litigation, to open up the channels for “illegal acts” being included in litigation, and to construct the criteria for determining “illegal acts” under different circumstances. The reason why it is advisable to adopt the system of “behavior standard as the main criterion and result standard as a supplement” is based on two considerations: first, it is conducive to playing the role of environmental laws and social roles. Environmental law, by providing for specific matters of prohibitive norms, guides people not to make a certain behavior, reducing the probability of illegal behavior and playing a guiding role in environmental law; at the same time, strict environmental laws are more favorable for promoting ecological civilization. Second, behavioral norms are more certain than the consequences of damage. Complete legal norms often contain assumptions, behavior patterns, and legal consequences of three elements with clear elements of legal norms. In judicial practice, the legal consequences of environmental pollution and damage are often difficult to be quantified as specific damage in a short period of time but are reflected as potential risks. For example, in the civil public interest lawsuit on ecological damage brought by Zhang and others mentioned above, the court believed that the damage caused by the defendant by climbing Monty Python Peak by inserting pitons was inevitable, but what was the extent of such damage? There is no legal forensic institution in the country that can make such a judgment. Therefore, it is more beneficial to implement the environmental protection concept of ecological protection to construct the standards of matters based on behavior standards.

#### 4.2.3. The Concept of “Prevention First, Remedy Second” Should Be Respected

Preventing environmental damage and ecological destruction is the primary goal of the system, and remedying the damage to the public due to illegal behavior is a secondary goal. This concept also includes the idea of dualization and the inclined allocation of judicial and administrative resources. In terms of dualism, this concept emphasizes that the risk of environmental degradation should be solved through the active intervention of administrative organs and normalized type of supervision, while at the same time, the infringers should be evaluated negatively through the litigation mechanism, and monetary compensation and environmental restoration should be provided for the existing damages. In terms of judicial and administrative resource allocation, the intention is to emphasize that the timing of environmental public interest litigation intervention should be before the damage is caused. In the face of environmental pollution and ecological damage, there are two different strategies for environmental public interest litigation: ex post remedial and ex ante prevention. Ex post remedial environmental public interest litigation focuses on the end point. However, this ex post approach is not fully effective in reducing or preventing environmental risks and cannot address the complexity, comprehensiveness, and irreversibility of environmental damage and ecological destruction. Modern environmental safety systems emphasize prevention and risk reduction in advance. In the face of the “uncertainty” of environmental risks, the prevention-oriented litigation mode is based on risk, breaks the traditional boundary of actual damage, and intervenes in advance when there is doubt, prediction, or concern about potential environmental hazards. This often relies on environmental supervision and management departments or other administrative organs taking the initiative to detect and prevent environmental pollution and ecological damage that has already occurred but has not yet caused serious consequences, as well as foreseeable environmental pollution and ecological damage that has not yet occurred but has great potential to occur. This system is designed to dovetail with China’s current public interest litigation mechanism, which will not only give full play to the supervision function of the procuratorial organs but also enhance the effectiveness of governance of the administrative organs.

## 5. Conclusions

Through the analysis of China’s legislative texts and judicial decisions, we can reach the following two conclusions: one is that the legal types and scope of application of environmental public interest litigation in China are constantly expanding; the other is that the scope of environmental public interest litigation in China mainly focuses on “environmental pollution” and “ecological damage”. Under the concept of building a beautiful China, it is necessary to eliminate all acts of environmental pollution and ecological damage as much as possible. To this end, we believe that the scope of the application of environmental public interest litigation should be expanded according to the law, which requires establishing certain mechanisms.

First, it is important to ensure a smooth connection between procuratorial recommendations and environmental administrative public interest litigation. As mentioned above, environmental administrative public interest litigation has its unique advantages in protecting the ecological environment, and China should focus on expanding the application of environmental administrative public interest litigation in the new era. The low application rate of environmental administrative public interest litigation in China’s judicial practice is closely related to the setting of the pre-litigation prosecution proposal system. According to the 2022 work report of the Supreme People’s Procuratorate of China, procuratorial organs filed 169,000 cases of public interest litigation, including 149,000 administrative public interest litigations. However, only 11,000 of these cases were filed, i.e., nearly 93% of the cases stayed in the pre-litigation prosecution stage. It can be seen that China uses its judicial status to protect the public interest before litigation and promote source management by consultation and pre-litigation procuratorial suggestions, causing the majority of cases to stay in the pre-litigation stage [54]. As a result, it is also natural to designate procuratorial recommendations as the best choice for environmental public interest litigation. A closer look reveals that there is an obstacle between the procuratorial recommendation and public interest litigation, that is, the administrative organs perform their duties in accordance with the standards of identification. To initiate environmental public interest litigation, the people’s procuratorate must prove that the prosecution meets the legal requirements and that the administrative organ does not correct the violation or perform its duties after the procuratorial recommendation is issued. In other words, in addition to proving that the national interests and social welfare have been infringed, it is also necessary to prove that the administrative organ has violated the law. In practice, it is difficult for the procuratorial authorities to define whether the administrative organ has violated the law or failed to perform its statutory duties, especially when it “should have acted but did not fully act”.

In this regard, we argue that China should first establish the criteria for determining administrative violations in environmental administrative public interest litigation. Only when we have clear criteria for administrative organs to perform their duties in accordance with the law can the procuratorial organs make professional judgments on whether to file environmental administrative public interest litigation. This will enable us to expand the scope of the application of environmental public interest litigation and urge environmental administrative departments to conscientiously perform their duties to protect the ecological environment.

Second, a guiding case library and a sound legal interpretation mechanism should be built. Even with the establishment of the standards of the principle of “behavior standard as the main criterion and result standard as a supplement” and “prevention-based, remediation-supplemented”, it is not possible to cover all the acts of environmental pollution and ecological damage in reality. The legislation cannot provide for the scope of all environmental public interest litigation, so we believe there is still room for further improvement in the legal interpretation mechanism. The ultimate goal of legal interpretation is to obtain a normative meaning, i.e., “to explore the standard meaning of the law in today’s legal order (its normative meaning today), which can only be determined by considering both the historical legislator’s intention and his specific normative idea, rather than ignoring it altogether” [55]. To ensure that the criteria for judging environmental public interest litigation matters established in this dissertation do not deviate from the original intention of the legislation, we argue that China can further improve the legal interpretation mechanism and effectively solve the problem of fuzzy standards for the scope of application of environmental public interest litigation in practice. At the same time, a mechanism can be built for discovering, cultivating, and recommending guiding cases, compiling typical cases of “environmental pollution” and “ecological damage”. In addition, we should give full play to the role of demonstration and guidance of typical cases to clarify and expand the scope of application of environmental public interest litigation.

Third, a mechanism should be established to link environmental protection organizations, people’s procuratorates, and environmental administrative departments. Since the beginning of environmental public interest litigation in 2012, the scope of the application of environmental public interest litigation in China has been under constant exploration, which is closely related to the fact that environmental public interest litigation is not independent of other public interest litigation, but lacks a unified interface mechanism between legal supervision and environmental public interest protection. First of all, environmental public interest litigation is different from other public interest litigation, and its internal system interface can be solved through a sound legal interpretation mechanism, such as broadening the scope of environmental civil public interest litigation through legal interpretation so that it is consistent with the scope of civil litigation. Second, risk prevention has become a common task among various state organs. With the deepening of China’s reform and opening up and the transformation of society, the deterioration of the ecological environment and the lack of natural resources are becoming more and more prominent. The responsibility of preventing and coping with environmental risks has accordingly become an urgent task for various state organs. This depends on establishing an external articulation mechanism for environmental public interest litigation. To this end, an information communication mechanism between the procuratorate, environmental administrative departments, and environmental organizations should be established to provide the procuratorate with a complete chain from the source of clues and process supervision to the feedback of results [56]. Subsequently, a consultation and implementation mechanism between the procuratorate and the environmental administration can be established, which makes the procuratorate effectively perform its legal supervision duties on the one hand, and urges the administration to perform its duties to protect the environment in accordance with the law on the other hand. This will realize the synergy between the procuratorate and the environmental administration in environmental public welfare protection.

## Data Availability

Not applicable.
